# Modulation of GSK-3 provides cellular and functional neuroprotection in the *rd10* mouse model of retinitis pigmentosa

**DOI:** 10.1186/s13024-018-0251-y

**Published:** 2018-04-16

**Authors:** Alonso Sánchez-Cruz, Beatriz Villarejo-Zori, Miguel Marchena, Josefa Zaldivar-Díez, Valle Palomo, Carmen Gil, Ignacio Lizasoain, Pedro de la Villa, Ana Martínez, Enrique J. de la Rosa, Catalina Hernández-Sánchez

**Affiliations:** 1 Departments of Molecular Biomedicine (3D Lab) and Structural and Chemical Biology (IPSBB Unit), Centro de Investigaciones Biológicas-CSIC, C/ Ramiro de Maeztu 9, E-28040 Madrid, Spain; 2 Neurovascular Research Unit, Department of Pharmacology, Facultad de Medicina, Universidad Complutense de Madrid, Madrid, Spain; 3 Department of Systems Biology, Facultad de Medicina, Universidad de Alcalá, Alcalá de Henares, Spain

**Keywords:** GSK-3, Retina, Retinitis pigmentosa, *rd10*, Neurodegeneration, Therapy

## Abstract

**Background:**

Retinitis pigmentosa (RP) is a group of hereditary retinal neurodegenerative conditions characterized by primary dysfunction and death of photoreceptor cells, resulting in visual loss and, eventually, blindness. To date, no effective therapies have been transferred to clinic. Given the diverse genetic etiology of RP, targeting common cellular and molecular retinal alterations has emerged as a potential therapeutic strategy.

**Methods:**

Using the *Pde6b*^*rd10/rd10*^ mouse model of RP, we investigated the effects of daily intraperitoneal administration of VP3.15, a small-molecule heterocyclic GSK-3 inhibitor. Gene expression was analyzed by quantitative PCR and protein expression and phosphorylation by Western blot. Photoreceptor preservation was evaluated by histological analysis and visual function was assessed by electroretinography.

**Results:**

In *rd10* retinas, increased expression of pro-inflammatory markers and reactive gliosis coincided with the early stages of retinal degeneration. Compared with wild-type controls, GSK-3β expression (mRNA and protein) remained unchanged during the retinal degeneration period. However, levels of GSK-3β^Ser9^ and its regulator Akt^Ser473^ were increased in *rd10* versus wild-type retinas. In vivo administration of VP3.15 reduced photoreceptor cell loss and preserved visual function. This neuroprotective effect was accompanied by a decrease in the expression of neuroinflammatory markers.

**Conclusions:**

These results provide proof of concept of the therapeutic potential of VP3.15 for the treatment of retinal neurodegenerative conditions in general, and RP in particular.

**Electronic supplementary material:**

The online version of this article (10.1186/s13024-018-0251-y) contains supplementary material, which is available to authorized users.

## Background

Inherited retinal dystrophies, which include retinitis pigmentosa (RP), are a group of genetic diseases caused by mutations in over 300 genes and loci (http://www.sph.uth.tmc.edu/Retnet/disease.htm). RP is a retinal neurodegenerative condition characterized by primary dysfunction and death of photoreceptor cells, resulting in vision loss and, ultimately, blindness [1]. Although several experimental therapies have advanced to clinical trials (https://clinicaltrials.gov/ct2/results?term=retinitis+pigmentosa&Search=Search), neither approved treatments nor preventive therapies for RP are currently available. Its status as a rare disease and its diverse genetic etiology emphasize the importance of identifying shared pathological mechanisms independent of the causative mutation. Targeting common cellular and molecular retinal responses to mutations could benefit a significant number of RP patients, as well as those with other retinal dystrophies without an exclusively genetic etiology (e.g., glaucoma, age-related macular degeneration, diabetic retinopathy). Recent studies suggest that retinal neurodegeneration is associated with a broad inflammatory response in the retina. This response appears to be mutation-independent and involves microglial activation, reactive macrogliosis, and the production of pro-inflammatory cytokines [2–5].

GSK-3 is a serine/threonine kinase that exists as 2 highly homologous isoforms, GSK-3α and GSK-3β, each encoded by a distinct gene. GSK-3β is predominantly expressed in the central nervous system (CNS) [6]. Although initially identified as a glycogen synthesis enzyme (the role for which it was named), GSK-3 is currently considered a multitask enzyme involved in the regulation of diverse cellular functions owing to its broad substrate spectrum [7, 8]. In particular, GSK-3 plays a pivotal role in regulating the balance between pro-inflammatory and anti-inflammatory cellular responses. Consequently, GSK-3 is considered a potential therapeutic target for diseases with an inflammatory component. The therapeutic potential of GSK-3 modulators is currently being studied in a variety of neuroinflammatory diseases. These include psychiatric and neurodegenerative disorders, as well as retinal dystrophies; as part of the CNS, the retina shares many physiological and pathological traits with the brain [9].

Employing a chemical genetic approach, we recently investigated the neuroprotective effects of 3 chemically diverse GSK-3 modulators in photoreceptor cells [10]. In the present study we selected one of these compounds, VP3.15, based on its favorable pharmacokinetic and IC_50_ properties. This molecule is a 5-imino-thiadiazole that has been described not only as substrate-competitive GSK-3 inhibitor [11], but also as allosteric inhibitor of PDE7 [12].

We sought to validate in vivo the potential of GSK-3 as a therapeutic target for the treatment of RP, and to demonstrate the therapeutic potential of VP3.15 in this neurodegenerative condition. We characterized the expression of GSK-3β and its inactive serine-9-phosphorylated form in the dystrophic retina of the *rd10* mouse, a model of RP. Moreover, we demonstrated that chronic systemic in vivo VP3.15 treatment preserved visual function by delaying neurodegeneration. Our results indicate that VP3.15 is an innovative drug candidate for the treatment of RP.

## Methods

### Animals and drug delivery

The *rd10* mouse model of retinal neurodegeneration carries a homozygous phosphodiesterase 6b mutation (*Pde6b*^*rd10/rd10*^) on a C57BL/6 J background. Mice were kindly provided by Bo Chang from The Jackson Laboratory (Bar Harbor, ME, USA). Wild type (WT) control mice of the same background were also obtained from The Jackson Laboratory. All animals were housed and handled in accordance with the ARVO statement for the Use of Animals in Ophthalmic and Vision Research, European Union guidelines, and those of the local ethics committees of the CSIC and the Comunidad de Madrid. Mice were bred at the CIB core facilities on a 12/12-h light/dark cycle.

VP3.15 was synthesized in our laboratory as previously described [11]. This small molecule is a member of the iminothiadiazole family, the first group of substrate-competitive GSK-3 inhibitors described ([11]; see Additional file 1: Figure S1 for the structure data). The dose administrated was selected based on pharmacokinetic and IC_50_ data ([11–13] and data not shown). Mice received daily intraperitoneal injections of vehicle (10% DMSO, 0.9% NaCl) or VP3.15 (10 mg/kg) for the indicated period of time.

IP administration was selected based on the ability of VP3.15 to cross the brain blood barrier [13] and the necessity of performing repetitive administration without damaging the eye.

### Electroretinography

Mice underwent electroretinographic recordings (ERG) at different time points, using a previously described ERG protocol [14]. ERGs were performed by an observer blind to treatment. ERG signals were amplified and band filtered between 0.3 Hz and 1000 Hz (CP511 Preamplifier, Grass Instruments, Quincy, MA, USA) and digitized at 10 kHz using a PowerLab acquisition data card (AD Instruments Ltd., Oxfordshire, UK). Graphical representations of the signals recorded and the control light stimuli were generated using Scope v6.4 PowerLab software. ERG wave amplitudes were measured off-line and the results averaged.

### Histology and immunostaining

Mice were euthanized at the indicated ages and their eyes enucleated. The right eye was processed for histological analyses and the left eye for retinal RNA extraction. For histological analyses, eyes were fixed for 1 h in 4% (*w*/*v*) paraformaldehyde in 0.1 M phosphate buffer (PB), pH 7.4, and then cryoprotected by incubation in increasing concentrations of sucrose (final concentration 50% (w/v) in PB). The eyes were then embedded in Tissue-Tek OCT (Sakura Finetec, Torrance, CA, USA) and frozen on isopropanol/dry ice. Cryostat equatorial sections (12 μm) were mounted on Superfrost® Plus slides (Thermo Scientific, Massachusetts, USA), dried at room temperature, and stored at − 20 °C. Before performing further analyses, sections were fixed in acetone for 10 min at − 20 °C and dried at 65 °C for 10 min. After rinsing in PBS and permeation with 1% (w/v) Triton X-100 in PBS, sections were blocked in BGT (2.5 g/L BSA, 100 mM glycine, 0.25% (w/v) Triton X-100 in PBS) for 1 h and then incubated overnight at 4 °C with primary antibodies (Table 1) diluted in BGT. Sections incubated in the absence of primary antibody were used as specificity controls. After rinsing in PBS and incubation with the appropriate secondary antibodies (Table 1), sections were stained with DAPI (4′,6-diamidino-2-phenylindole; Sigma-Aldrich Corp., St. Louis, MO, USA) and coverslipped with Fluoromont-G.

**Table 1:** Antibodies

Antibody against	Host species	Dilution	Manufacturer	Catalog number
Rhodopsin	Mouse	IH,1:500	Abcam, Cambridge, UK	AB3267
L/M-opsin	Rabbit	IH,1:200	Abcam	AB5405
Mouse-Igs, Rabbit-Igs Alexa 488-546-647 labeled	Goat	IH, 1:200–500	ThermoFisher Scientific, Waltham, MA	A-11001
				A-11008
				A-11004
				A-11011
				A-21235
GSK-3Ββ	Mouse	IH,1:100	Cell Signaling Technology, Danvers, Massachusetts, USA	9832S
pGSK-3Ββ^S9^	Mouse	IH,1:400	Cell Signaling Technology	93,235
Recoverin	Rabbit	WB,1:5000	Millipore, Billerica, Massachusetts, USA	AB5585
GSK-3Ββ	Mouse	WB,1:1000	Cell Signaling Technology	9832S
pGSK-3Ββ^S9^	Rabbit	WB,1:1000	Cell Signaling Technology	93,235
Akt	Rabbit	WB,1:1000	Cell Signaling Technology	9272
pAkt^S473^	Rabbit	WB,1:1000	Cell Signaling Technology	9271 L
pAkt^S473^	Rabbit	IH,1:100	Cell Signaling Technology	4060
GFAP	Rabbit	IH, 1:500	Dako, Glostrup (Denmark)	Z0334

*IH* immunohistochemistry, *WB* Western blot

To assess preservation of the photoreceptor layer, we compared the thickness of the ONL (which primarily contains photoreceptors) with that of the corresponding INL (which contains bipolar, horizontal, and amacrine neurons and Müller glial cell bodies), and quantified the length of rod and cone outer segments (OS). Three sections per eye were analyzed: for each section, one image was captured for each of the 6 retinal zones (T1, T2, T3, T4, T5, and T6; Additional file 2: Figure S2). In each image, 3 measurements were recorded at random positions to obtain an average value per retinal zone per section. Measurements were performed using the “freehand line” and “measure” tools in Fiji software. The ONL thickness was normalized to that of the INL (not affected by the degeneration at this stage) to correct for possible inclinations of the sectioning plane.

### Immunoblots

Protein extraction was carried out by sonicating individual retinas in RIPA lysis buffer (containing 2 mM Na_3_VO_4_, 10 mM NaF, and 4 mM Na_4_P_2_O_7_) and chilling the resulting solution on ice for 30 min. Protein (30 μg) from each sample was fractionated by electrophoresis on precast 10–12% (*w*/*v*) SDS-polyacrylamide gels (Criterion TGX, Bio-Rad, Munich, Germany), after which proteins were transferred to PVDF membranes using the Trans-Blot Turbo system (Bio-Rad). Blots were incubated overnight at 4 °C with primary antibodies (Table 1) diluted in TBS (Tris-buffered saline) containing 1% (w/v) Triton-X100, followed by incubation with the appropriate peroxidase-conjugated secondary antibody (Table 1). Proteins were visualized using the Pierce® ECL Western Blotting Substrate (ThermoFisher Scientific, Waltham, MA, USA), and quantified using ChemiDoc™ Touch Imaging System (Bio-Rad).

### RNA isolation and quantitative PCR

Total RNA from individual retinas was extracted using TRIzol Reagent, and 2.5 μg of RNA was typically reverse transcribed using the Superscript III Kit and random primers (all from ThermoFisher Scientific). Quantitative PCR (qPCR) was performed with the ABI Prism 7900HT Sequence Detection System using TaqMan Universal PCR Master Mix, no-AmpErase UNG, and Taqman assays (listed below) for detection (all from Thermo Fisher). The relative change in gene expression was calculated using the 2^ΔCt^ method, normalizing to expression levels of the *Tbp* (TATA-binding protein) gene. The primer-probe sets used are listed in Table 2.

**Table 2:** Taqman assays

Gene	Taqman assay
*α2m*	Mm00558642_m1
*Gfap*	Mm01253033_m1
*Il1β*	Mm00434228_m1
*Tbp*	Mm00446971_m1
*Tnfα*	Mm00443260_g1
*Rho*	Mm01184405_m1
*Iba1*	Mm00479862_g1
*Cd68*	Mm03047343_m1
*Cd11b*	Mm00434455_m1

### Statistical analysis

Statistical analyses were performed with GraphPad Prism software 6.0 (GraphPad Software Inc., La Jolla, CA, USA). Protein levels were compared using an unpaired t-test. Gene expression levels were compared using either one-way or 2-way ANOVA tests as indicated in the Figure Legend. For ERG data, differences in wave amplitudes over time between vehicle- and VP3.15-treated mice were assessed using a 2-way ANOVA. Histological differences between vehicle- and VP3.15-treated mice were assessed by 2-way ANOVA. In all cases, *p*-values ≤0.05 were considered statistically significant.

## Results

### Inflammatory genes are upregulated in rd10 mice during the early stages of retinal neurodegeneration

**Fig. 1:**
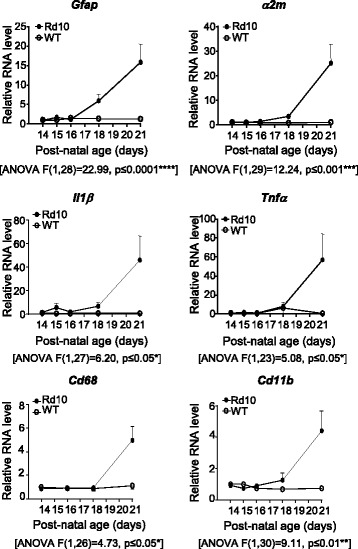
Expression of inflammatory genes in the retina. RT-qPCR of WT and *rd10* retinas at the indicated ages. Levels of different transcripts were normalized to those of *Tbp* RNA and relativized to P14 WT level (= 1). Results represent the mean + SEM. *n* = 3–4, *p ≤ 0.05, **p ≤ 0. 01, ***p ≤ 0.001, **** *p* ≤ 0.0001 (2-way ANOVA)

Pathological neuroinflammation is a hallmark of neurodegeneration in many retinal dystrophies [2–5]. We analyzed temporal alterations in pro-inflammatory gene expression in the *rd10* retina between P14 (before the appearance of morphological signs of retinal degeneration) and P21 (at which stage the degenerative process has become clearly established) to identify the optimal period for therapeutic intervention. Quantitative PCR analysis of WT and *rd10* retinas revealed increased expression of the pro-inflammatory markers *α2m*, *Il1β* and *Tnfα* in the degenerating retinas, beginning in the early stages of degeneration (P18) and increasing dramatically (by 25 to 50 fold) by P21 (Fig. 1). Reactive gliosis in response to retinal damage, as measured by upregulated *Gfap* expression, correlated with the increased expression of inflammatory markers. Likewise, the expression of the microglia genes *Cd68* and *Cd11b* also showed a 4–5 fold increase (Fig. 1).

### GSK-3β expression in wild type and rd10 retinas

**Fig. 2:**
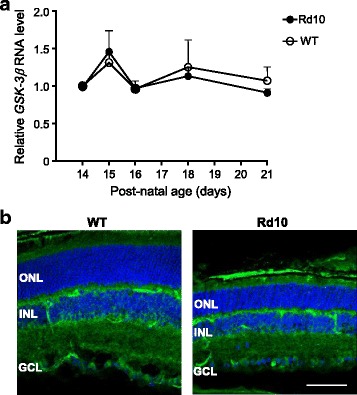
GSK-3β expression in the retina. **a** RT-qPCR of WT and *rd10* retinas at the indicated ages. *Gsk-3β* transcript levels were normalized to *Tbp* RNA levels and relativized to P14 WT level (= 1). Results represent the mean + SEM. *n* = 4. **b** Representative images of P21 retinal sections from WT and *rd10* mice, immunostained for GSK-3β (green). Nuclei are stained with DAPI (blue). ONL, outer nuclear layer; INL, inner nuclear layer; GCL, ganglion cell layer. Scale bar: 60 μm

We next determined expression levels of GSK-3β, the most abundant GSK-3 isoform in the CNS [6], during the same degenerative period as above. We measured the expression of total GSK-3β and of its inactive Ser9-phosphorylated form (GSK-3β^Ser9^) in *rd10* mouse retinas. No significant differences in GSK-3β RNA levels were observed between *rd10* and WT retinas (Fig. 2a). Furthermore, despite the morphological differences caused by the degenerative process, we observed similarly broad distribution patterns for GSK-3β protein in both P21 *rd10* and WT retinas (Fig. 2b).

**Fig. 3:**
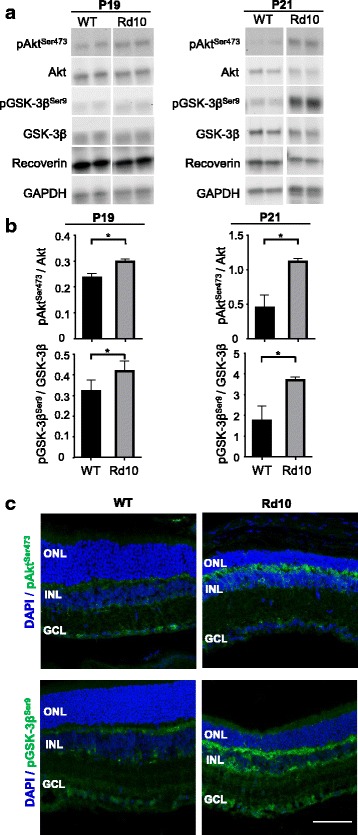
GSK-3β activation in the retina. **a** Representative Western blots of protein extracts from WT and *rd10* retinas (at P19 and P21). **b** Densitometric analysis of several membranes as those shown in **a**. Levels of phosphorylated Akt and GSK-3β were normalized to those of total Akt and GSK-3β, respectively. Results represent the mean + SEM. *n* = 4–5, *p ≤ 0.05 (unpaired 2-tailed Student’s t test). **c** Representative images of P21 retinal sections from WT and *rd10* mice, immunostained for pAkt^Ser473^ and pGSK-3β^Ser9^ (green). Nuclei are stained with DAPI (blue). ONL, outer nuclear layer; INL, inner nuclear layer; GCL, ganglion cell layer. Scale bar: 60 μm

GSK-3β is an unusual kinase in that it is constitutively activated but is inhibited upon stimulation of its regulatory signaling pathways (reviewed in [7]). Activation of the PI3K/Akt pathway induces phosphorylation of GSK-3β at Ser9, and subsequent downregulation of GSK-3β activity [15]. During early retinal degeneration (P16 and P18), no differences in Ser^473^-phosphorylated Akt (pAkt^Ser473^) or Ser^9^-phosphorylated GSK-3β (pGSK-3β^Ser9^) levels were observed between WT and *rd10* retinas (data not shown). Conversely, at P19 and P21, at which point the degenerative process is well established, as evidenced by decreased expression of the photoreceptor protein recoverin, pAkt^Ser473^ and pGSK-3β^Ser9^ levels were significantly higher in *rd10* versus WT retinas (Fig. 3a and b). These observations in retinal extracts were in agreement with the immunostaining pattern observed at P21, which showed an increment in pAkt^Ser473^ and pGSK-3β^Ser9^ in the *rd10* retina (Fig. 3c). This inhibitory phosphorylation of GSK-3β, which coincided with activation of the pro-survival Akt signaling pathway, may represent an intrinsic neuroprotective response to the genetic damage caused in the retina. To test this hypothesis, we sought to potentiate this intrinsic response by pharmacologically inhibiting GSK-3 activity.

### In vivo VP3.15 treatment decreases inflammatory and degenerative signs in the rd10 mouse retina

**Fig. 4:**
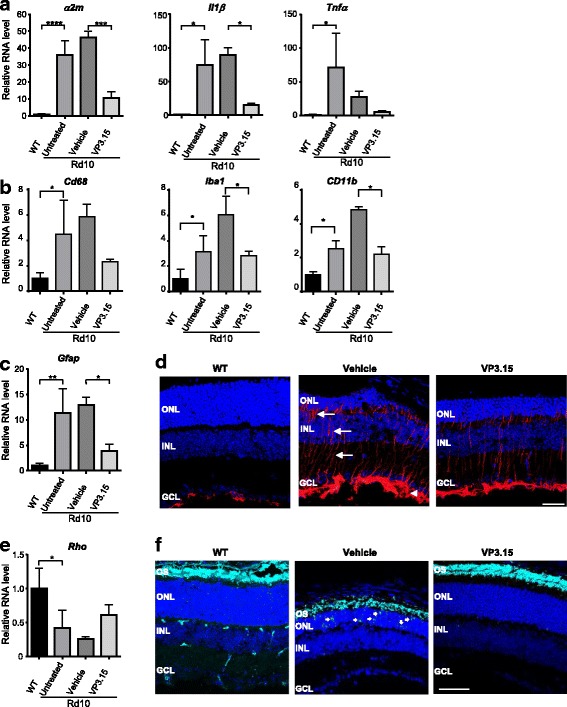
In vivo effect of short-term VP3.15 administration. *rd10* mice received daily intraperitoneal injections of vehicle or VP3.15 from P15 to P21, and were analyzed one day after the last injection (P22). **a-c** and **e** RT-qPCR of WT as well as untreated *rd10* retinas, and vehicle- and VP3.15-treated *rd10* retinas (all harvested at P22). Levels of all transcripts were normalized to those of *Tbp* RNA and relativized to WT levels (= 1). Results represent the mean + SEM. *n* = 2–4, *p ≤ 0.05, **p ≤ 0.01, ***p ≤ 0.001, **** *p* ≤ 0.0001 (one-way ANOVA). **d** and **f** Representative images of P22 retinal sections from WT mice and from vehicle- and VP3.15-treated *rd10* mice, immunostained for GFAP (**d**, red) and rhodopsin (**f**, cyan). Nuclei are stained with DAPI. In **d** arrows point to the radial processes of Müller cells and the arrow head to the most inner retinal layer containing the Müller end feet and astrocytes. Arrows in **f** point to mislocalized rhodopsin in the ONL. OS, outer segments; ONL, outer nuclear layer; INL, inner nuclear layer; GCL, ganglion cell layer. Scale bar: 60 μm

In light of our findings concerning proinflammatory gene expression and GSK-3β modulation, we selected the P15–P21 interval to assess the effects of short-term treatment with VP3.15, a GSK-3 inhibitor. VP3.15 inhibitory activity on GSK-3 was confirmed by determining its effect on the β-catenin levels in the microglia cell line N9 (Additional file 3: Figure S3). *rd10* littermates received daily intraperitoneal injections of either vehicle or VP3.15 (10 mg/kg). One eye from each animal was analyzed for gene expression and the other for retinal morphology. VP3.15 treatment significantly decreased the expression of the proinflammatory genes *Il1β* and *α2m*. A similar trend was observed for *Tnfα* expression (Fig. 4a), and secretion by *rd10* retinal explants (Additional file 7: Figure S7b), although these failed to reach statistical significance. The microglia genes *Cd11b* and *Iba1* were also decreased upon VP3.15 treatment, while the activated microglia marker *Cd68* showed a decline trend (Fig. 4b). In parallel, VP3.15 significantly reduced the expression of *Gfap*, an effect that correlates with a reduced GFAP staining depicted by the radial process of Müller glial cells and by the most inner retinal layer formed by Müller end feet and astrocytes (Fig. 4c and d). Conversely, a trend towards increased gene expression of rod photoreceptor-specific rhodopsin was observed in VP3.15-treated *rd10* mice (Fig. 4e). This observation correlated with the maintenance of ONL thickness and OS length, both of which were better preserved in VP3.15-treated than in the vehicle-treated *rd10* mice (Fig. 4f). Moreover, while rhodopsin expression was specifically localized in the photoreceptor outer segment (OS) in WT and VP3.15-treated *rd10* retinas, vehicle-treated *rd10* retinas displayed shortening of the OS and mislocalization of rhodopsin in the ONL (Fig. 4f).

### In vivo treatment of rd10 mice with VP3.15 delays the loss of visual function

**Fig. 5:**
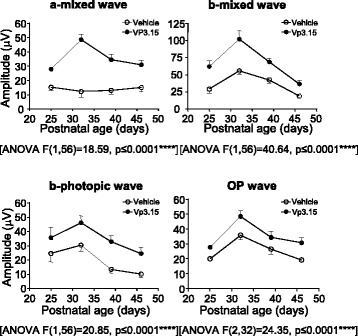
In vivo effect of long-term VP3.15 administration on visual function. *rd10* mice received daily intraperitoneal injections of vehicle or VP3.15 from P15 to P45. ERG were performed at the indicated ages. Graphs show averaged ERG wave amplitudes, plotted as a function of animal age. Results represent the mean ± SEM. *n* = 10, **** *p* ≤ 0.0001 (2-way ANOVA)

Based on the promising results obtained following short-term GSK-3 inhibition, we investigated whether a longer treatment period preserved visual function in the *rd10* mouse model. *rd10* littermates received daily injections of either vehicle or VP3.15 from P15 to P46 inclusive. Visual function was assessed weekly by ERG between P25 and P46. Compared with vehicle-treated counterparts, VP3.15-treated *rd10* mice displayed better-defined ERG waves of greater amplitude (Fig. 5 and Additional file 4: Figure S4). The amplitude of ERG waves (a-mixed, b-mixed, b-photopic, and oscillatory potential [OP]) was significantly higher in VP3.15-treated than in vehicle-treated mice, indicating a prolongation of both rod- and cone-mediated vision function by VP3.15 (Fig. 5).

**Fig. 6:**
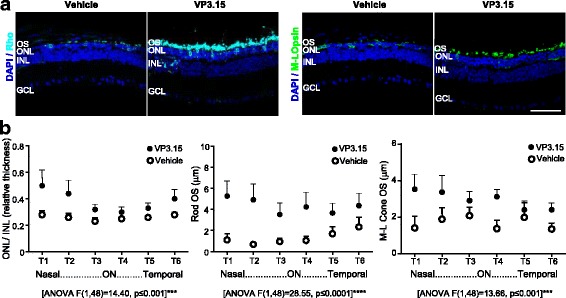
In vivo effect of long-term VP3.15 administration on photoreceptor cell preservation. Retinal sections from the same animals described in Fig. 5 were immunostained to assess photoreceptor cell structure. **a** Representative images of P47 retinal sections from vehicle- and VP3.15-treated *rd10* mice immunostained for rhodopsin (cyan) and M/L opsin (green). Nuclei are stained with DAPI (blue). OS, outer segments; ONL, outer nuclear layer; INL, inner nuclear layer; GCL, ganglion cell layer. Scale bar: 60 μm. **b** ONL and INL thickness and the length of rod and cone outer segments were measured in equatorial sections corresponding to 6 regions of the retina, following a nasotemporal sequence (T1–T6; see Methods and Additional file 2: Figure S2). Plots show the mean + SEM. *n* = 5 mice, 3 sections per retina, 3 measurements per region and section. ON, optic nerve. ***p ≤ 0.001, **** *p* ≤ 0.0001 (2-way ANOVA)

Histological evaluation of retinas at the end point of the study (P47, after the last ERG) revealed a greater relative ONL thickness in VP3.15- versus vehicle-treated retinas despite the advanced degeneration stage (Fig. 6a and b). The photoreceptor preservation effect of VP3.15 was more evident at mid-degeneration stages (P33; Additional file 5: Figure S5). Moreover, specific immunostaining showed better preservation of photoreceptor structure in VP3.15-treated retinas (Fig. 6a). Rhodopsin immunostaining in rod outer segments confirmed the partial preservation of rod outer segments in VP3.15-treated retinas. By contrast, rod outer segments were barely present in vehicle-treated retinas. Similarly, M-L opsin immunostaining revealed longer cone outer segments in VP3.15- versus vehicle-treated *rd10* retinas (Fig. 6a and b).

Moreover, no toxic effect of VP3.15 administration was observed in other neuronal populations of the retina, particularly rod-bipolar and ganglion cells (Additional file 6: Figure S6).

Taken together, these results demonstrate a neuroprotective effect of VP3.15 on neuroinflammation, retinal structure, and visual function. Our findings thus support the potential of VP3.15 as a novel therapy for retinal dystrophies.

## Discussion

This study describes GSK-3β expression and activation of the Akt-GSK-3β pathway during the early stages of retinal neurodegeneration in the *rd10* mouse model of RP. Our findings support the involvement of GSK-3 in retinal decay and provide proof of concept of the neuroprotective effect of GSK-3 inhibition on photoreceptor cells [10]. In vivo administration of VP3.15, a small-molecule GSK-3 inhibitor, reduced photoreceptor cell loss and extended visual function. This neuroprotective effect was accompanied by a decrease in the expression of neuroinflammatory genes in the retina, indicating the potential of VP3.15 as a candidate RP therapy.

GSK-3 is a constitutively activated multitask enzyme, the activity of which is regulated by several inhibitory signaling pathways [7]. Inhibitory phosphorylation of GSK-3 at Ser9 is one of the main regulatory mechanisms. Our analysis of GSK-3β RNA and protein levels during the degenerative period in *rd10* retinas revealed no differences with respect to WT counterparts. However, at both P19 and P21, by when photoreceptor degeneration is well established, inhibitory phosphorylation of GSK-3β at Ser9, and phosphorylation of its Akt regulator, were increased in *rd10* retinas. This endogenous activation of the prosurvival Akt-GSK-3β pathway in the *rd10* retina is in agreement with previous observations in a photoreceptor cell line subjected to a variety of insults, and in the dystrophic retina [16, 17]. We speculate that the activation of endogenous prosurvival pathways in the dystrophic retina constitutes an attempt to control the degenerative process, a response that, however, results insufficient to counteract the permanent genetic damage underlying the degeneration. In our experiments, administration of the GSK-3 inhibitor VP3.15 may have exogenously potentiated the prosurvival pathway, thus delaying photoreceptor cell death and preserving visual function. Indeed, several agents with neuroprotective effects in the retina exert common stimulatory effects on the Akt-GSK-3β pathway [18–20]. GSK-3 is a downstream substrate of the insulin receptor [15, 21] and is thus inhibited upon insulin receptor stimulation. We have previously demonstrated that proinsulin, which binds with high affinity to the A isoform of the insulin receptor in the retina and activates Akt [22, 23], similarly prolongs rod survival and preserves visual function [14, 22, 24].

Neuroinflammation is a key component of neurodegenerative processes affecting the brain and retina, and thus constitutes a promising non-cell-autonomous target for treatments of CNS diseases in general, and of the retina in particular [2–5, 25]. GSK-3 plays a pivotal role in the regulation of peripheral and central pro-inflammatory cytokine production [26, 27], and its inhibition reduces both systemic and brain inflammation [26, 27]. The specific molecular events that occur in the *rd10* retina upon GSK-3 inhibition remain to be established. This is a complicated task since GSK-3 is implicated in the regulation of multiple cellular processes including metabolism, cell structure, cell death, proliferation, and gene expression, with over 100 confirmed and 500 predicted substrates [7]. Exploratory studies employing *rd10* retinal explants showed that VP3.15 attenuated NF-kB activation (Additional file 7: Figure S7). NF-kB is a key regulator of the inflammatory response by enabling the transcription of the genes encoding many pro-inflammatory cytokines. Indeed, our results suggest that levels of pro-inflammatory cytokines (*Il1β* and *Tnfα*) are reduced in VP3.15-treated *rd10* retinas. This could explain, at least in part, the attenuation of photoreceptor degeneration and loss, and the preservation of vision. Moreover, the reduced inflammatory response observed in VP3.15-treated retinas was accompanied by decreases in the reactive gliosis marker GFAP as well as in *α2M* expression, for which a role in retinal neurodegeneration has been recently described [28–30].

In addition to its inhibitory effect on GSK-3, VP3.15 also acts as an allosteric inhibitor of PDE7, an effect that contributes to its neuroprotective activity in other CNS pathologies [13]. However, PDE7 inhibition, via increasing cAMP levels and PKA activation, also converges on GSK-3 inhibition [31]. PKA is able to phosphorylate GSK-3β at Ser9 and, subsequently, to inactivate it [20, 31]. Besides, previous findings by our group support that the prosurvival effect of VP3.15 in photoreceptors may be primarily due to GSK-3 inhibition, since the specific GSK-3 inhibitor tideglusib exerted an even more potent neuroprotective effect in photoreceptors [10].

Most forms of RP involve primary death of rod photoreceptors, in which the mutated gene exerts its function, followed by secondary loss of cone photoreceptors. VP3.15 treatment prolonged photoreceptor survival, as evidenced by the preservation of ONL thickness and rod outer segment length. Furthermore, VP3.15 preserved the integrity of the cone outer segment, the first site of morphological alterations during cone degeneration. Human vision primarily relies on cones, which mediate daylight, color, and high-acuity vision. Accordingly, a mutation-independent treatment for RP that specifically prolongs cone survival would be highly beneficial. VP3.15 had a marked effect on cone cytoarchitecture and visual function, as indicated by an increase in the amplitude of the photopic b-wave compared with the untreated *rd10* animals. It remains unclear whether VP3.15 exerts a direct effect on cone integrity, or whether this effect is secondary to the prosurvival effect on rods (present study and [10]). Regardless, VP3.15 resulted in a marked attenuation of the loss of both daylight and dim-light vision.

## Conclusion

Our in vivo findings, together with the results of a recently published in vitro study [10], support the therapeutic potential of GSK-3 modulation for the treatment of retinal neurodegeneration, as described for other neurodegenerative diseases [32], and underscore the potential of GSK-3 inhibitors, in particular VP3.15, as pharmacological therapies for RP.

## Additional files

Additional file 1: **Figure S1.** Chemical structure of VP3.15. (PPTX 49 kb)

Additional file 2: **Figure S2.** Scheme of the retinal sections. The 6 retinal zones defined for quantification as T1, T2, T3, T4, T5 and T6 are indicated. ON, optic nerve. (PPTX 593 kb)

Additional file 3: **Figure S3.** Effect of VP3.15 on β-catenin levels. N9 microglia cell cultures were treated either with vehicle or with 10 μM VP3.15 for 1 or 7 h. **a** Representative Western blots of protein extracts from cultured N9 cells at the indicated times. **b** Densitometric analysis of membranes as those shown in **a**. Levels of β-catenin were normalized to those of GAPDH. Results represent the mean + SEM. *n* = 3, *p ≤ 0.05 (unpaired 2-tailed Student’s t test). Methods are provided in Additional file 8. (PPTX 11435 kb)

Additional file 4: **Figure S4.** VP3.15 treated mice show better light-evoked responses than vehicle-treated ones. Standard ERG representative trace recordings from one VP3.15-treated and one vehicle-treated overnight dark-adapted animal. See for comparison the differences between the two experimental groups in the trace amplitudes. Rod and cone mixed response (a-mixed and b-mixed, 1.5 log cd·s/m^2^), and oscillatory potential (OP, 1.5 log cd·s/m^2^) were recorded sequentially under scotopic conditions. Cone (b-phot, 2 log cd·s/m^2^) responses were recorded after 5 min light-adaptation (30 cd/m^2^ background light) under photopic conditions. All light responses were separated in the vertical axis to better present the ERG waveform. Animal age is indicated to the left of each trace recording. (PPTX 1167 kb)

Additional file 5: **Figure S5.** Mid-term effect of VP3.15 on photoreceptor preservation. *rd10* mice received daily an intraperitoneal injection of vehicle or VP3.15 from P15 to P32 and the retinas were analyzed one day after the last injection (P33). Representative images of P33 retinal sections from WT and vehicle- and VP3.15-treated *rd10* mice stained with DAPI (blue). ONL, outer nuclear layer; INL, inner nuclear layer. Scale bar: 60 μm. **b** ONL and INL thickness were measured in equatorial sections corresponding to 6 regions of the retina, following a nasotemporal sequence (T1–T6; see Methods and Additional file 2: Figure S2). Plot shows the mean + SEM. *n* = 3 mice, 3 sections per retina, 3 measurements per region and section. ON, optic nerve. ****p ≤ 0.0001 (2-way ANOVA). (PPTX 360 kb)

Additional file 6: **Figure S6.** In vivo effect of VP3.15 treatment on rod-bipolar and ganglion cells. *rd10* mice received daily an intraperitoneal injection of vehicle or VP3.15 from P15 to P32 and the retinas were analyzed one day after the last injection (P33). **a** Representative images of P33 retinal sections from vehicle- and VP3.15-treated *rd10* mice, immunostained for PKCα or RBPMS to label rod-bipolar or ganglion cells respectively (green). Nuclei are stained with DAPI (blue). **b** The number of PKCα- and RBPMS-positive cells were scored in equatorial sections corresponding to 6 regions of the retina, following a nasotemporal sequence (T1–T6; see Methods and Additional file 2: Figure S2). The plots show the mean + SEM. n = 3 mice, 3 sections per retina, 3 measurements per region and section. ONL, outer nuclear layer; INL, inner nuclear layer; GCL, ganglion cell layer. Scale bar: 38 μm. Methods are provided in Additional file 8. (PPTX 924 kb)

Additional file 7: **Figure S7.** Effect of VP3.15 on NF-kB activation and TNFα secretion. P22 rd10 retinas were cultured in the absence (vehicle) or presence of 3.2 μM VP3.15 for 16 h. **a** Representative images showing Western blots of protein extracts. **b** Densitometric analysis of membranes as those shown in **a**. Levels of pNF-kB^Ser536^ were normalized to those of GAPDH. Results represent the mean + SEM. *n* = 5–6, *p ≤ 0.05 (unpaired 2-tailed Student’s t test). **c** TNFα concentration was quantified in the culture media by ELISA. Methods are provided in Additional file 8. (PPTX 6297 kb)

Additional file 8: Additional Methods. (DOCX 20 kb)

## Abbreviations

CD11B: Cluster of differentiation molecule 11B
CD68: Cluster of differentiation 68
CNS: Central nervous system
ERG: Electroretinographic recording
GCL: Ganglion cell layer
GFAP: Glial fibrillary acidic protein
GSK: Glycogen synthase kinase
IBA1: Ionized calcium-binding adapter molecule 1
IC_50_: Half maximal inhibitory concentration
IL1-β: Interleukin-1β
INL: Inner nuclear layer
NF-κB: Nuclear factor kappa-light-chain-enhancer of activated B cells
ON: Optic nerve
ONL: Outer nuclear layer
OP: Oscillatory potential
OS: Outer segment
PB: Phosphate buffer
PDE: Phosphodiesterase
PKA: Protein kinase A
PKCα: Protein kinase C alpha
qPCR: Quantitative polymerase chain reaction
RBPMS: RNA binding protein with multiple splicing
RP: Retinitis pigmentosa
RT: Reverse transcription
TBP: TATA-binding protein
TNFα: Tumor necrosis factorα
WT: Wild-type
α2M: α-2-macroglobulin

## References

[1] Hartong DT, Berson EL, Dryja TP (2006). Retinitis pigmentosa. Lancet.

[2] Chinskey ND, Besirli CG, Zacks DN (2014). Retinal cell death and current strategies in retinal neuroprotection. Curr Opin Ophthalmol.

[3] Cuenca N, Fernandez-Sanchez L, Campello L, Maneu V, De la Villa P, Lax P, Pinilla I (2014). Cellular responses following retinal injuries and therapeutic approaches for neurodegenerative diseases. Prog Retin Eye Res.

[4] Mustafi D, Maeda T, Kohno H, Nadeau JH, Palczewski K (2012). Inflammatory priming predisposes mice to age-related retinal degeneration. J Clin Invest.

[5] Yoshida N, Ikeda Y, Notomi S, Ishikawa K, Murakami Y, Hisatomi T, Enaida H, Ishibashi T (2013). Clinical evidence of sustained chronic inflammatory reaction in retinitis pigmentosa. Ophthalmology.

[6] Pardo M, Abrial E, Jope RS, Beurel E (2016). GSK3beta isoform-selective regulation of depression, memory and hippocampal cell proliferation. Genes Brain Behav.

[7] Beurel E, Grieco SF, Jope RS (2015). Glycogen synthase kinase-3 (GSK3): regulation, actions, and diseases. Pharmacol Ther.

[8] Jope RS, Johnson GV (2004). The glamour and gloom of glycogen synthase kinase-3. Trends Biochem Sci.

[9] Jindal V (2015). Interconnection between brain and retinal neurodegenerations. Mol Neurobiol.

[10] Marchena M, Villarejo-Zori B, Zaldivar-Diez J, Palomo V, Gil C, Hernandez-Sanchez C, Martinez A, de la Rosa EJ (2017). Small molecules targeting glycogen synthase kinase 3 as potential drug candidates for the treatment of retinitis pigmentosa. J Enzyme Inhib Med Chem.

[11] Palomo V, Perez DI, Perez C, Morales-Garcia JA, Soteras I, Alonso-Gil S, Encinas A, Castro A, Campillo NE, Perez-Castillo A (2012). 5-imino-1,2,4-thiadiazoles: first small molecules as substrate competitive inhibitors of glycogen synthase kinase 3. J Med Chem.

[12] Redondo M, Palomo V, Brea J, Perez DI, Martin-Alvarez R, Perez C, Paul-Fernandez N, Conde S, Cadavid MI, Loza MI (2012). Identification in silico and experimental validation of novel phosphodiesterase 7 inhibitors with efficacy in experimental autoimmune encephalomyelitis mice. ACS Chem Neurosci.

[13] Medina-Rodriguez EM, Bribian A, Boyd A, Palomo V, Pastor J, Lagares A, Gil C, Martinez A, Williams A, de Castro F (2017). Promoting in vivo remyelination with small molecules: a neuroreparative pharmacological treatment for multiple sclerosis. Sci Rep.

[14] Corrochano S, Barhoum R, Boya P, Arroba AI, Rodriguez-Muela N, Gomez-Vicente V, Bosch F, de Pablo F, de la Villa P, de la Rosa EJ (2008). Attenuation of vision loss and delay in apoptosis of photoreceptors induced by proinsulin in a mouse model of retinitis pigmentosa. Invest Ophthalmol Vis Sci.

[15] Cross DA, Alessi DR, Cohen P, Andjelkovich M, Hemmings BA (1995). Inhibition of glycogen synthase kinase-3 by insulin mediated by protein kinase B. Nature.

[16] Finnegan S, Mackey AM, Cotter TG (2010). A stress survival response in retinal cells mediated through inhibition of the serine/threonine phosphatase PP2A. Eur J Neurosci.

[17] Nakazawa T, Shimura M, Tomita H, Akiyama H, Yoshioka Y, Kudou H, Tamai M (2003). Intrinsic activation of PI3K/Akt signaling pathway and its neuroprotective effect against retinal injury. Curr Eye Res.

[18] Baek SM, Yu SY, Son Y, Hong HS (2016). Substance P promotes the recovery of oxidative stress-damaged retinal pigmented epithelial cells by modulating Akt/GSK-3beta signaling. Mol Vis.

[19] Rajala A, Gupta VK, Anderson RE, Rajala RV (2013). Light activation of the insulin receptor regulates mitochondrial hexokinase. A possible mechanism of retinal neuroprotection. Mitochondrion.

[20] Wyse Jackson AC, Cotter TG (2016). The synthetic progesterone Norgestrel is neuroprotective in stressed photoreceptor-like cells and retinal explants, mediating its effects via basic fibroblast growth factor, protein kinase A and glycogen synthase kinase 3beta signalling. Eur J Neurosci.

[21] van Weeren PC, de Bruyn KM, de Vries-Smits AM, van Lint J, Burgering BM (1998). Essential role for protein kinase B (PKB) in insulin-induced glycogen synthase kinase 3 inactivation. Characterization of dominant-negative mutant of PKB. J Biol Chem.

[22] Isiegas C, Marinich-Madzarevich JA, Marchena M, Ruiz JM, Cano MJ, de la Villa P, Hernandez-Sanchez C, de la Rosa EJ, de Pablo F (2016). Intravitreal injection of proinsulin-loaded microspheres delays photoreceptor cell death and vision loss in the rd10 mouse model of retinitis Pigmentosa. Invest Ophthalmol Vis Sci.

[23] Malaguarnera R, Sacco A, Voci C, Pandini G, Vigneri R, Belfiore A (2012). Proinsulin binds with high affinity the insulin receptor isoform a and predominantly activates the mitogenic pathway. Endocrinology.

[24] Fernandez-Sanchez L, Lax P, Isiegas C, Ayuso E, Ruiz JM, de la Villa P, Bosch F, de la Rosa EJ, Cuenca N (2012). Proinsulin slows retinal degeneration and vision loss in the P23H rat model of retinitis pigmentosa. Hum Gene Ther.

[25] Ransohoff RM (2016). How neuroinflammation contributes to neurodegeneration. Science.

[26] Beurel E, Michalek SM, Jope RS (2010). Innate and adaptive immune responses regulated by glycogen synthase kinase-3 (GSK3). Trends Immunol.

[27] Jope RS, Cheng Y, Lowell JA, Worthen RJ, Sitbon YH, Beurel E (2017). Stressed and inflamed, can GSK3 be blamed?. Trends Biochem Sci.

[28] Barcelona PF, Saragovi HU (2015). A pro-nerve growth factor (proNGF) and NGF binding protein, alpha2-macroglobulin, differentially regulates p75 and TrkA receptors and is relevant to neurodegeneration ex vivo and in vivo. Mol Cell Biol.

[29] Shi Z, Rudzinski M, Meerovitch K, Lebrun-Julien F, Birman E, Di Polo A, Saragovi HU (2008). Alpha2-macroglobulin is a mediator of retinal ganglion cell death in glaucoma. J Biol Chem.

[30] Platon-Corchado M, Barcelona PF, Jmaeff S, Marchena M, Hernandez-Pinto AM, Hernandez-Sanchez C, Saragovi HU, de la Rosa EJ (2017). p75 NTR antagonists attenuate photoreceptor cell loss in murine models of retinitis pigmentosa. Cell Death Dis.

[31] Morales-Garcia JA, Palomo V, Redondo M, Alonso-Gil S, Gil C, Martinez A, Perez-Castillo A (2014). Crosstalk between phosphodiesterase 7 and glycogen synthase kinase-3: two relevant therapeutic targets for neurological disorders. ACS Chem Neurosci.

[32] Eldar-Finkelman H, Martinez A (2011). GSK-3 inhibitors: preclinical and clinical focus on CNS. Front Mol Neurosci.

