# Supplementary figures and images for: Modulation of GSK-3 provides cellular and functional neuroprotection in the rd10 mouse model of retinitis pigmentosa
Source: Mol Neurodegener. 2018 Apr 16;13:19. doi: 10.1186/s13024-018-0251-y (PMC5902946; doi:10.1186/s13024-018-0251-y)

## Slide 1
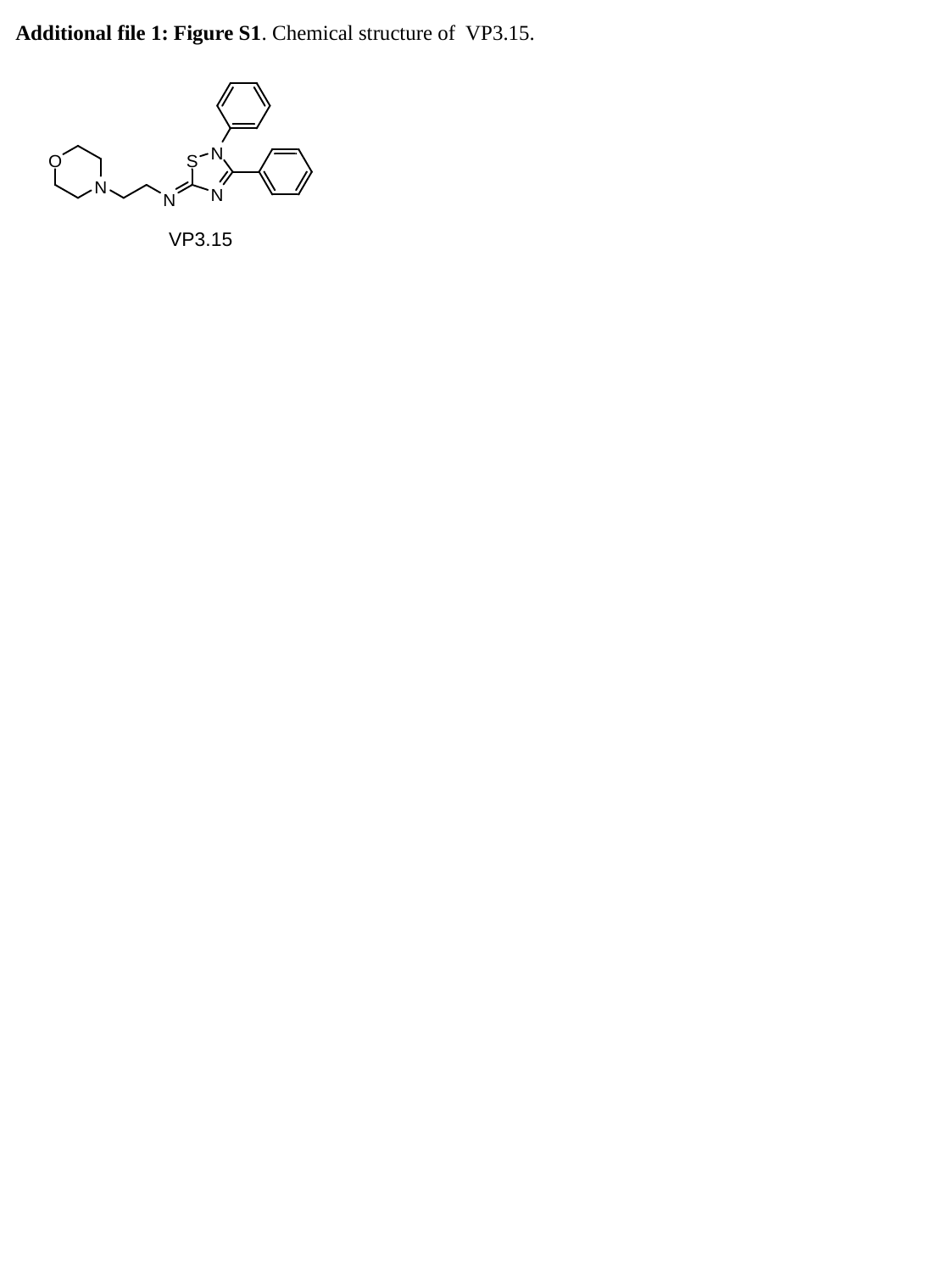

Additional file 1: Figure S1. Chemical structure of VP3.15.
VP3.15

Supplement: Supplementary file 1 — Figure S1. Chemical structure of VP3.15. (PPTX 49 kb) [file 13024_2018_251_MOESM1_ESM.pptx]
