# Supplementary material for: Modulation of GSK-3 provides cellular and functional neuroprotection in the rd10 mouse model of retinitis pigmentosa
Source: Mol Neurodegener. 2018 Apr 16;13:19. doi: 10.1186/s13024-018-0251-y (PMC5902946; doi:10.1186/s13024-018-0251-y)
Supplement: Supplementary file 2 — Figure S2. Scheme of the retinal sections. The 6 retinal zones defined for quantification as T1, T2, T3, T4, T5 and T6 are indicated. ON, optic nerve. (PPTX 593 kb) [file 13024_2018_251_MOESM2_ESM.pptx]

## Slide 1
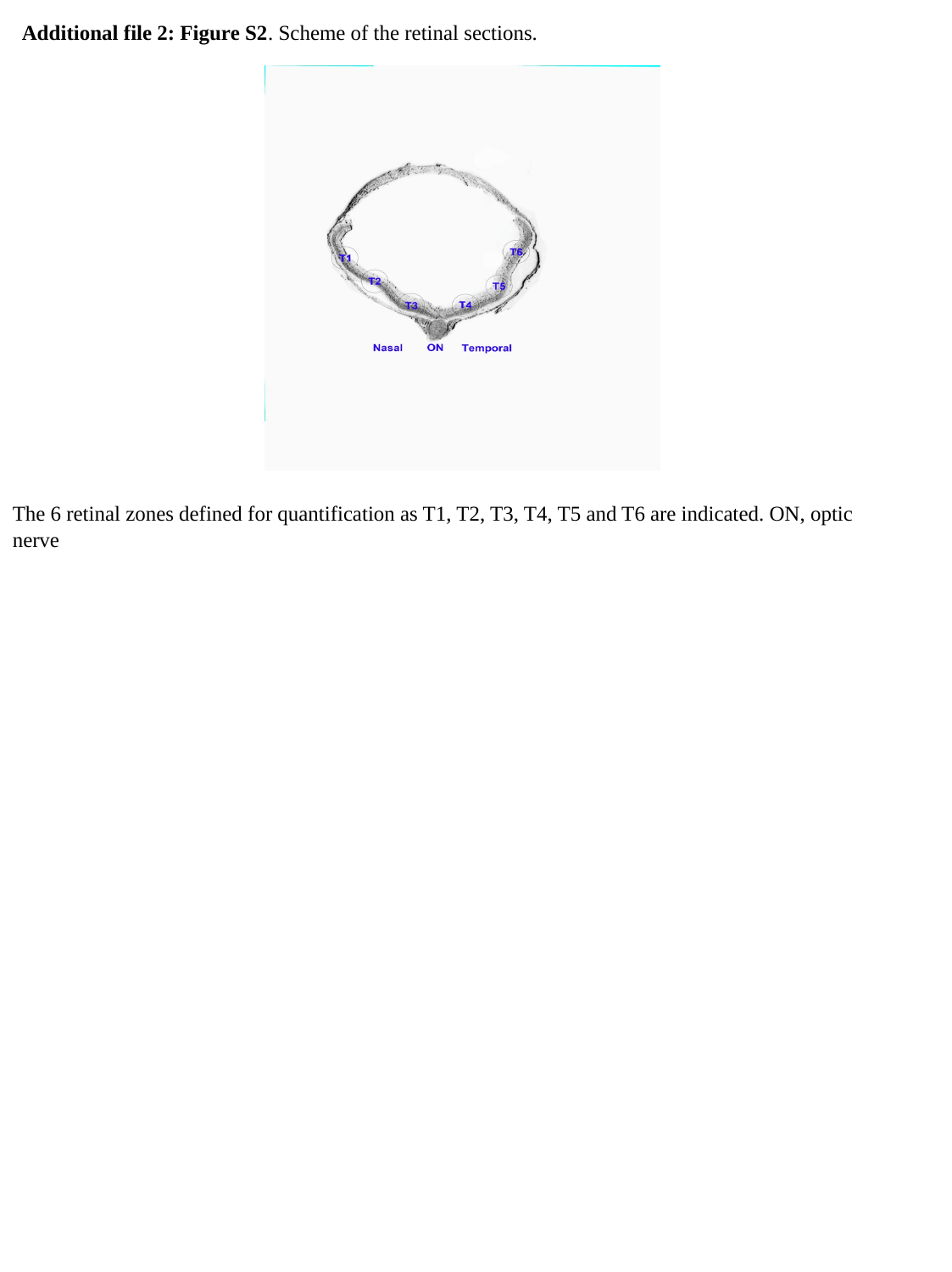

Additional file 2: Figure S2. Scheme of the retinal sections.
The 6 retinal zones defined for quantification as T1, T2, T3, T4, T5 and T6 are indicated. ON, optic nerve
