# Supplementary material for: Modulation of GSK-3 provides cellular and functional neuroprotection in the rd10 mouse model of retinitis pigmentosa
Source: Mol Neurodegener. 2018 Apr 16;13:19. doi: 10.1186/s13024-018-0251-y (PMC5902946; doi:10.1186/s13024-018-0251-y)
Supplement: Supplementary file 3 — Figure S3. Effect of VP3.15 on β-catenin levels. N9 microglia cell cultures were treated either with vehicle or with 10 μM VP3.15 for 1 or 7 h. a Representative Western blots of protein extracts from cultured N9 cells at the indicated times. b Densitometric analysis of membranes as those shown in a. Levels of β-catenin were normalized to those of GAPDH. Results represent the mean + SEM. n = 3, *p ≤ 0.05 (unpaired 2-tailed Student’s t test). Methods are provided in Additional file 8. (PPTX 11435 kb) [file 13024_2018_251_MOESM3_ESM.pptx]

## Slide 1
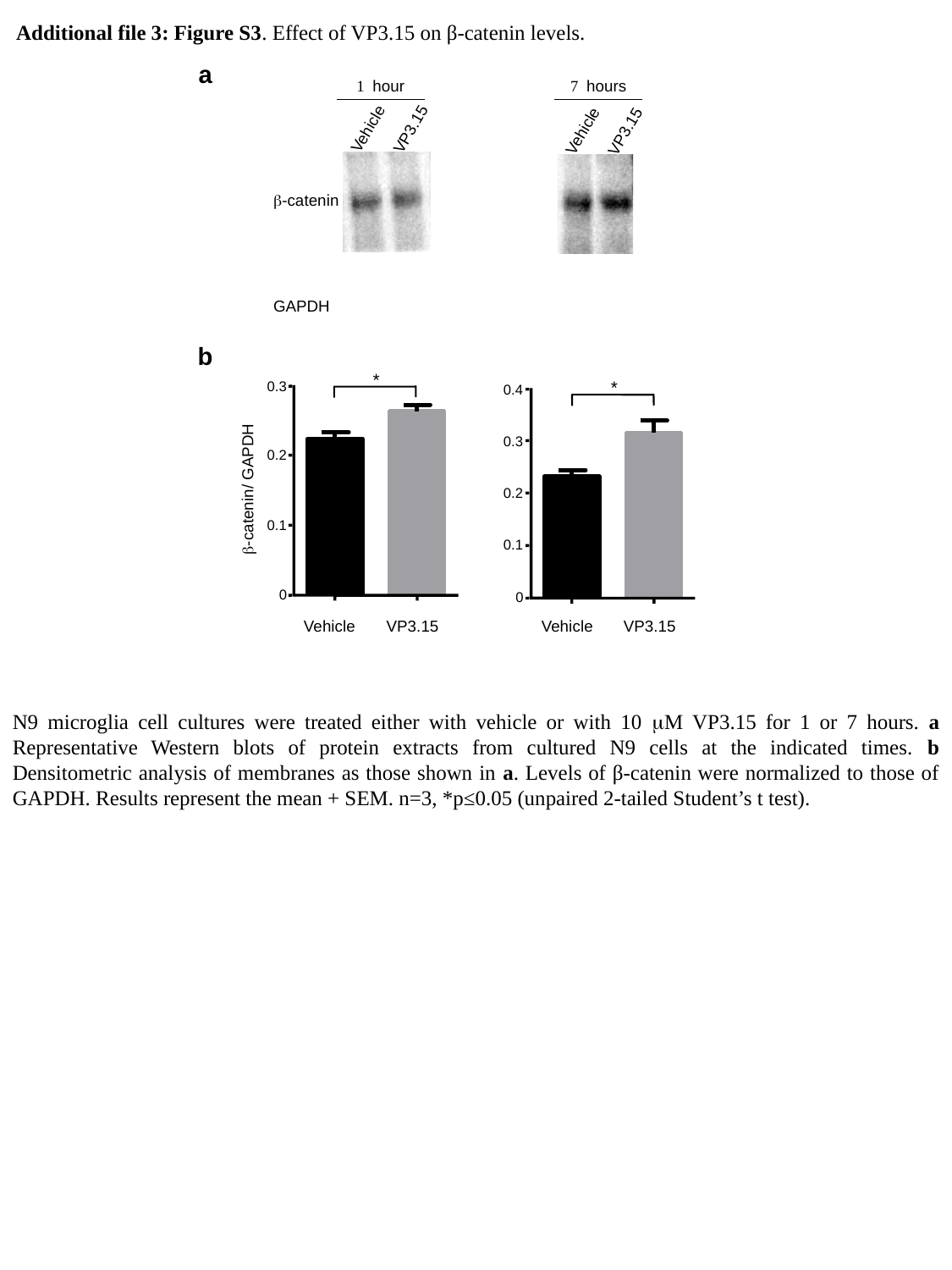

Additional file 3: Figure S3. Effect of VP3.15 on β-catenin levels.
a
1 hour
Vehicle
VP3.15
b-catenin
GAPDH
7 hours
Vehicle
VP3.15
b
*
0.3
0.2
b-catenin/ GAPDH
0.1
0
Vehicle
VP3.15
*
0.4
0.3
0.2
0.1
0
Vehicle
VP3.15
N9 microglia cell cultures were treated either with vehicle or with 10 mM VP3.15 for 1 or 7 hours. a Representative Western blots of protein extracts from cultured N9 cells at the indicated times. b Densitometric analysis of membranes as those shown in a. Levels of β-catenin were normalized to those of GAPDH. Results represent the mean + SEM. n=3, *p≤0.05 (unpaired 2-tailed Student’s t test).
