# Supplementary material for: Modulation of GSK-3 provides cellular and functional neuroprotection in the rd10 mouse model of retinitis pigmentosa
Source: Mol Neurodegener. 2018 Apr 16;13:19. doi: 10.1186/s13024-018-0251-y (PMC5902946; doi:10.1186/s13024-018-0251-y)
Supplement: Supplementary file 4 — Figure S4. VP3.15 treated mice show better light-evoked responses than vehicle-treated ones. Standard ERG representative trace recordings from one VP3.15-treated and one vehicle-treated overnight dark-adapted animal. See for comparison the differences between the two experimental groups in the trace amplitudes. Rod and cone mixed response (a-mixed and b-mixed, 1.5 log cd·s/m2), and oscillatory potential (OP, 1.5 log cd·s/m2) were recorded sequentially under scotopic conditions. Cone (b-phot, 2 log cd·s/m2) responses were recorded after 5 min light-adaptation (30 cd/m2 background light) under photopic conditions. All light responses were separated in the vertical axis to better present the ERG waveform. Animal age is indicated to the left of each trace recording. (PPTX 1167 kb) [file 13024_2018_251_MOESM4_ESM.pptx]

## Slide 1
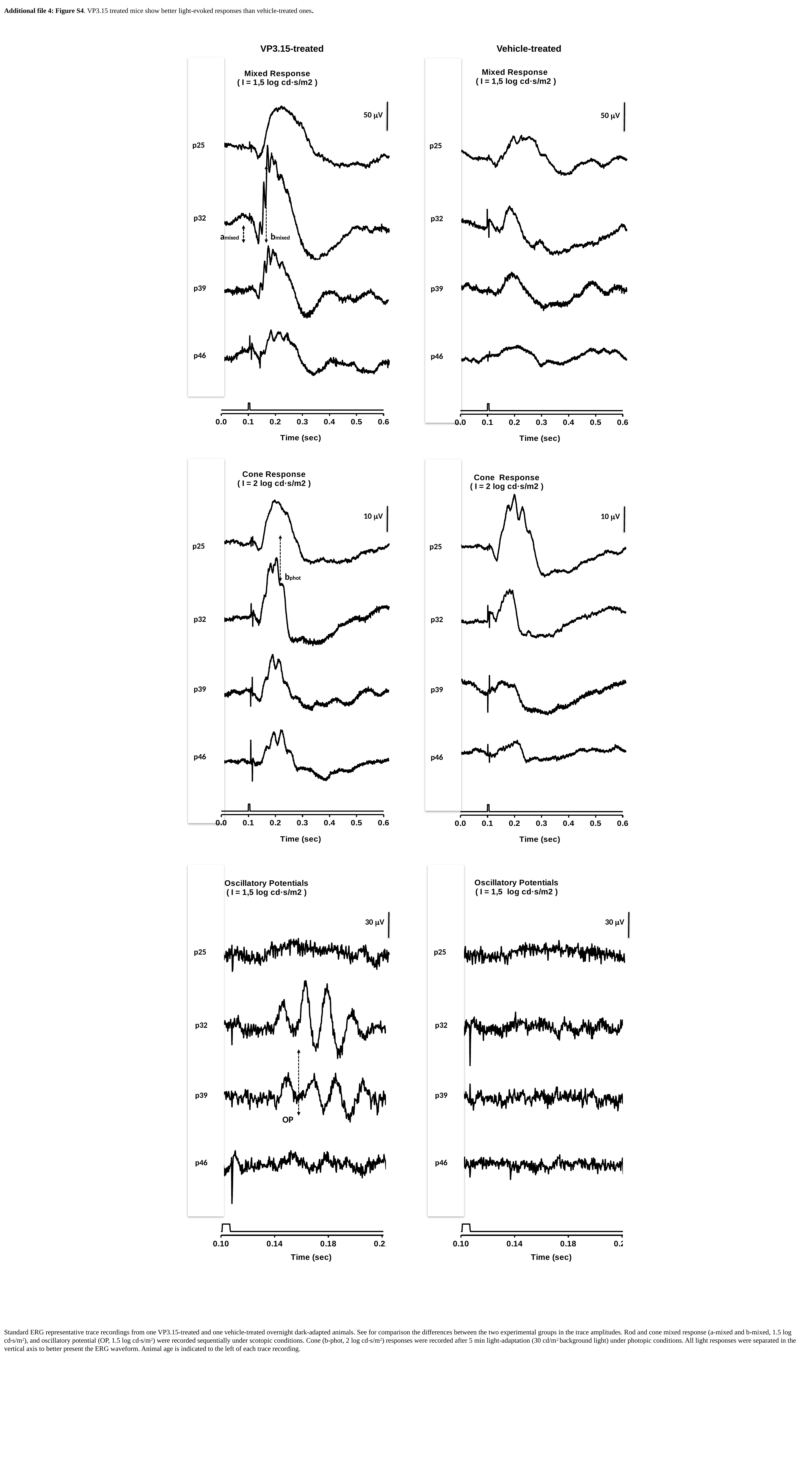

Additional file 4: Figure S4. VP3.15 treated mice show better light-evoked responses than vehicle-treated ones.
### Chart: Mixed Response
( I = 1,5 log cd·s/m2 )
| Category | 3.- Mixed ( I = 7 V) |
|---|---|
### Chart
| Category | 3.- Mixed ( I = 7 V) |
|---|---|50 mV
p25
### Chart
| Category | 3.- Mixed ( I = 7 V) |
|---|---|p32
### Chart
| Category | 3.- Mixed ( I = 7 V) |
|---|---|p39
p46
### Chart
| Category | |
|---|---|VP3.15-treated
Vehicle-treated
### Chart: Mixed Response
 ( I = 1,5 log cd·s/m2 )
| Category | 3.- Mixed ( I = 7 V) |
|---|---|
50 mV
### Chart
| Category | 3.- Mixed ( I = 7 V) |
|---|---|p25
### Chart
| Category | 3.- Mixed ( I = 7 V) |
|---|---|p32
### Chart
| Category | 2.- Rod ( I = 4 V) |
|---|---|p39
p46
### Chart
| Category | |
|---|---|amixed
bmixed
### Chart: Cone Response
( I = 2 log cd·s/m2 )
| Category | 5.- Conos ( I = 7 V) |
|---|---|
10 mV
### Chart
| Category | 5.- Conos ( I = 7 V) |
|---|---|p25
### Chart
| Category | 5.- Conos ( I = 7 V) |
|---|---|p32
### Chart
| Category | 5.- Conos ( I = 7 V) |
|---|---|p39
p46
### Chart
| Category | |
|---|---|
### Chart: Cone Response
( I = 2 log cd·s/m2 )
| Category | 5.- Conos ( I = 7 V) |
|---|---|
10 mV
### Chart
| Category | 5.- Conos ( I = 7 V) |
|---|---|p25
### Chart
| Category | 5.- Conos ( I = 7 V) |
|---|---|p32
### Chart
| Category | 5.- Conos ( I = 7 V) |
|---|---|p39
p46
### Chart
| Category | |
|---|---|bphot
### Chart: Oscillatory Potentials
( I = 1,5 log cd·s/m2 )
| Category | 4.- OP ( I = 7 V) |
|---|---|
30 mV
### Chart
| Category | 4.- OP ( I = 7 V) |
|---|---|p25
### Chart
| Category | 4.- OP ( I = 7 V) |
|---|---|p32
### Chart
| Category | 4.- OP ( I = 7 V) |
|---|---|p39
p46
### Chart
| Category | |
|---|---|
### Chart: Oscillatory Potentials
( I = 1,5 log cd·s/m2 )
| Category | 4.- OP ( I = 7 V) |
|---|---|
30 mV
### Chart
| Category | 4.- OP ( I = 7 V) |
|---|---|p25
### Chart
| Category | 4.- OP ( I = 7 V) |
|---|---|p32
### Chart
| Category | 4.- OP ( I = 7 V) |
|---|---|p39
p46
### Chart
| Category | |
|---|---|
OP
Standard ERG representative trace recordings from one VP3.15-treated and one vehicle-treated overnight dark-adapted animals. See for comparison the differences between the two experimental groups in the trace amplitudes. Rod and cone mixed response (a-mixed and b-mixed, 1.5 log cd·s/m2), and oscillatory potential (OP, 1.5 log cd·s/m2) were recorded sequentially under scotopic conditions. Cone (b-phot, 2 log cd·s/m2) responses were recorded after 5 min light-adaptation (30 cd/m2 background light) under photopic conditions. All light responses were separated in the vertical axis to better present the ERG waveform. Animal age is indicated to the left of each trace recording.
