# Supplementary material for: Modulation of GSK-3 provides cellular and functional neuroprotection in the rd10 mouse model of retinitis pigmentosa
Source: Mol Neurodegener. 2018 Apr 16;13:19. doi: 10.1186/s13024-018-0251-y (PMC5902946; doi:10.1186/s13024-018-0251-y)
Supplement: Supplementary file 5 — Figure S5. Mid-term effect of VP3.15 on photoreceptor preservation. rd10 mice received daily an intraperitoneal injection of vehicle or VP3.15 from P15 to P32 and the retinas were analyzed one day after the last injection (P33). Representative images of P33 retinal sections from WT and vehicle- and VP3.15-treated rd10 mice stained with DAPI (blue). ONL, outer nuclear layer; INL, inner nuclear layer. Scale bar: 60 μm. b ONL and INL thickness were measured in equatorial sections corresponding to 6 regions of the retina, following a nasotemporal sequence (T1–T6; see Methods and Additional file 2: Figure S2). Plot shows the mean + SEM. n = 3 mice, 3 sections per retina, 3 measurements per region and section. ON, optic nerve. ****p ≤ 0.0001 (2-way ANOVA). (PPTX 360 kb) [file 13024_2018_251_MOESM5_ESM.pptx]

## Slide 1
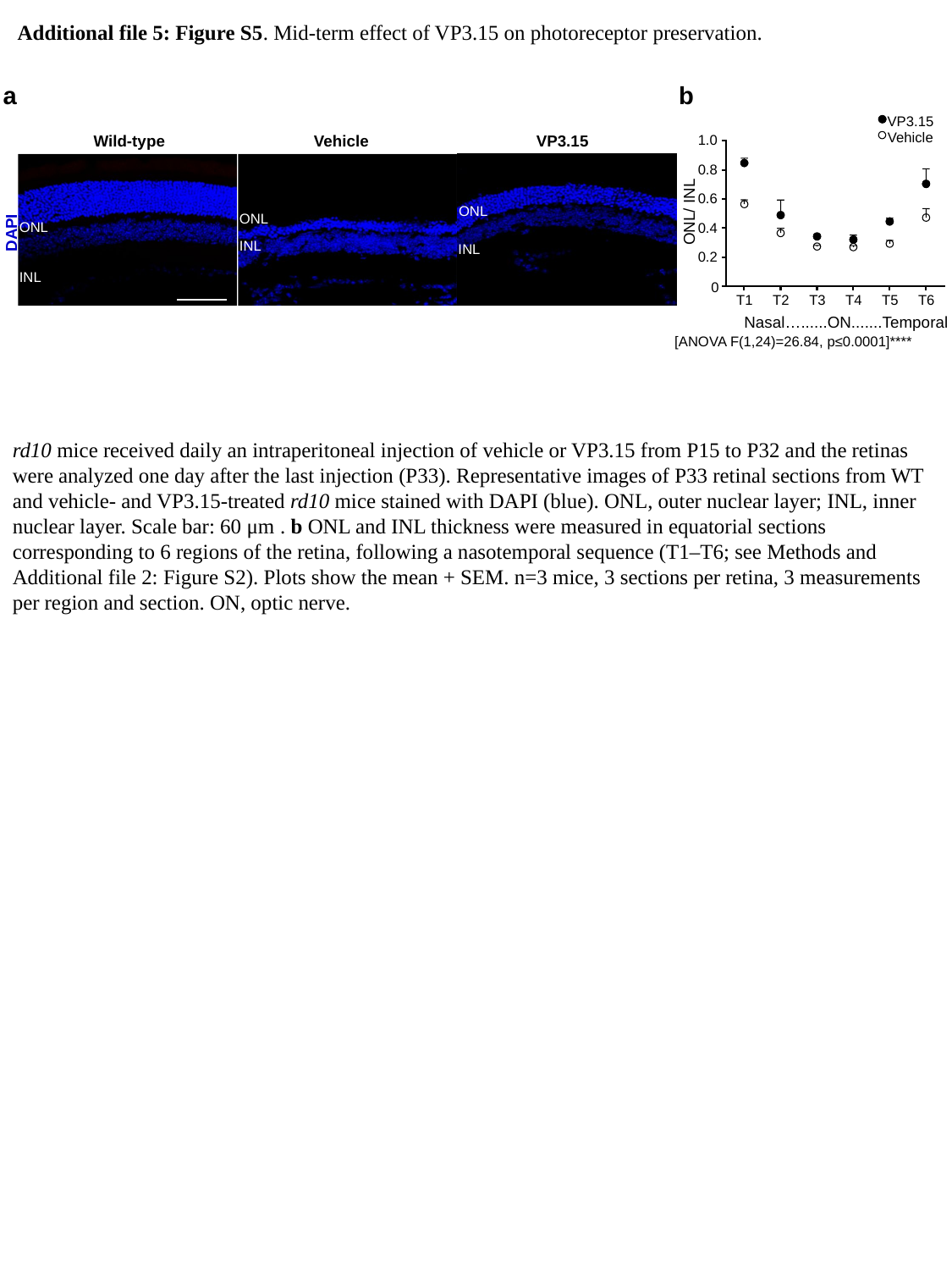

Additional file 5: Figure S5. Mid-term effect of VP3.15 on photoreceptor preservation.
a
b
VP3.15
Vehicle
1.0
0.8
0.6
ONL/ INL
0.4
0.2
0
T1
T2
T3
T4
T5
T6
Nasal…......ON.......Temporal
[ANOVA F(1,24)=26.84, p≤0.0001]****
Wild-type
Vehicle
VP3.15
ONL
ONL
ONL
DAPI
INL
INL
INL
rd10 mice received daily an intraperitoneal injection of vehicle or VP3.15 from P15 to P32 and the retinas were analyzed one day after the last injection (P33). Representative images of P33 retinal sections from WT and vehicle- and VP3.15-treated rd10 mice stained with DAPI (blue). ONL, outer nuclear layer; INL, inner nuclear layer. Scale bar: 60 μm . b ONL and INL thickness were measured in equatorial sections corresponding to 6 regions of the retina, following a nasotemporal sequence (T1–T6; see Methods and Additional file 2: Figure S2). Plots show the mean + SEM. n=3 mice, 3 sections per retina, 3 measurements per region and section. ON, optic nerve.
