# Supplementary material for: Modulation of GSK-3 provides cellular and functional neuroprotection in the rd10 mouse model of retinitis pigmentosa
Source: Mol Neurodegener. 2018 Apr 16;13:19. doi: 10.1186/s13024-018-0251-y (PMC5902946; doi:10.1186/s13024-018-0251-y)
Supplement: Supplementary file 6 — Figure S6. In vivo effect of VP3.15 treatment on rod-bipolar and ganglion cells. rd10 mice received daily an intraperitoneal injection of vehicle or VP3.15 from P15 to P32 and the retinas were analyzed one day after the last injection (P33). a Representative images of P33 retinal sections from vehicle- and VP3.15-treated rd10 mice, immunostained for PKCα or RBPMS to label rod-bipolar or ganglion cells respectively (green). Nuclei are stained with DAPI (blue). b The number of PKCα- and RBPMS-positive cells were scored in equatorial sections corresponding to 6 regions of the retina, following a nasotemporal sequence (T1–T6; see Methods and Additional file 2: Figure S2). The plots show the mean + SEM. n = 3 mice, 3 sections per retina, 3 measurements per region and section. ONL, outer nuclear layer; INL, inner nuclear layer; GCL, ganglion cell layer. Scale bar: 38 μm. Methods are provided in Additional file 8. (PPTX 924 kb) [file 13024_2018_251_MOESM6_ESM.pptx]

## Slide 1
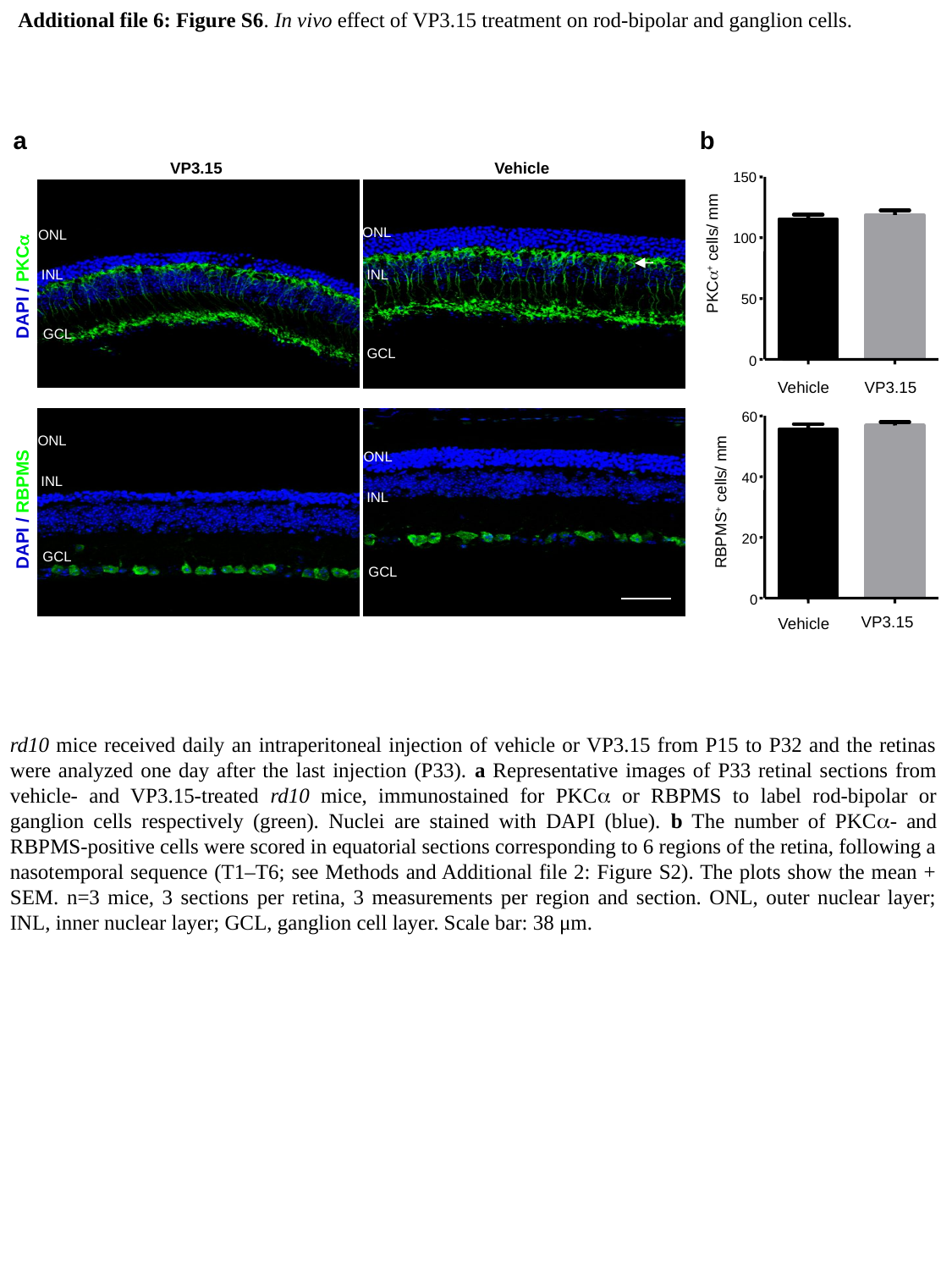

Additional file 6: Figure S6. In vivo effect of VP3.15 treatment on rod-bipolar and ganglion cells.
a
b
VP3.15
Vehicle
ONL
ONL
INL
INL
GCL
GCL
ONL
ONL
INL
INL
GCL
GCL
150
100
50
0
Vehicle
VP3.15
PKCa+ cells/ mm
DAPI / PKCa
60
40
20
0
VP3.15
Vehicle
RBPMS+ cells/ mm
DAPI / RBPMS
rd10 mice received daily an intraperitoneal injection of vehicle or VP3.15 from P15 to P32 and the retinas were analyzed one day after the last injection (P33). a Representative images of P33 retinal sections from vehicle- and VP3.15-treated rd10 mice, immunostained for PKCα or RBPMS to label rod-bipolar or ganglion cells respectively (green). Nuclei are stained with DAPI (blue). b The number of PKCα- and RBPMS-positive cells were scored in equatorial sections corresponding to 6 regions of the retina, following a nasotemporal sequence (T1–T6; see Methods and Additional file 2: Figure S2). The plots show the mean + SEM. n=3 mice, 3 sections per retina, 3 measurements per region and section. ONL, outer nuclear layer; INL, inner nuclear layer; GCL, ganglion cell layer. Scale bar: 38 μm.
