# Supplementary material for: Modulation of GSK-3 provides cellular and functional neuroprotection in the rd10 mouse model of retinitis pigmentosa
Source: Mol Neurodegener. 2018 Apr 16;13:19. doi: 10.1186/s13024-018-0251-y (PMC5902946; doi:10.1186/s13024-018-0251-y)
Supplement: Supplementary file 7 — Figure S7. Effect of VP3.15 on NF-kB activation and TNFα secretion. P22 rd10 retinas were cultured in the absence (vehicle) or presence of 3.2 μM VP3.15 for 16 h. a Representative images showing Western blots of protein extracts. b Densitometric analysis of membranes as those shown in a. Levels of pNF-kBSer536 were normalized to those of GAPDH. Results represent the mean + SEM. n = 5–6, *p ≤ 0.05 (unpaired 2-tailed Student’s t test). c TNFα concentration was quantified in the culture media by ELISA. Methods are provided in Additional file 8. (PPTX 6297 kb) [file 13024_2018_251_MOESM7_ESM.pptx]

## Slide 1
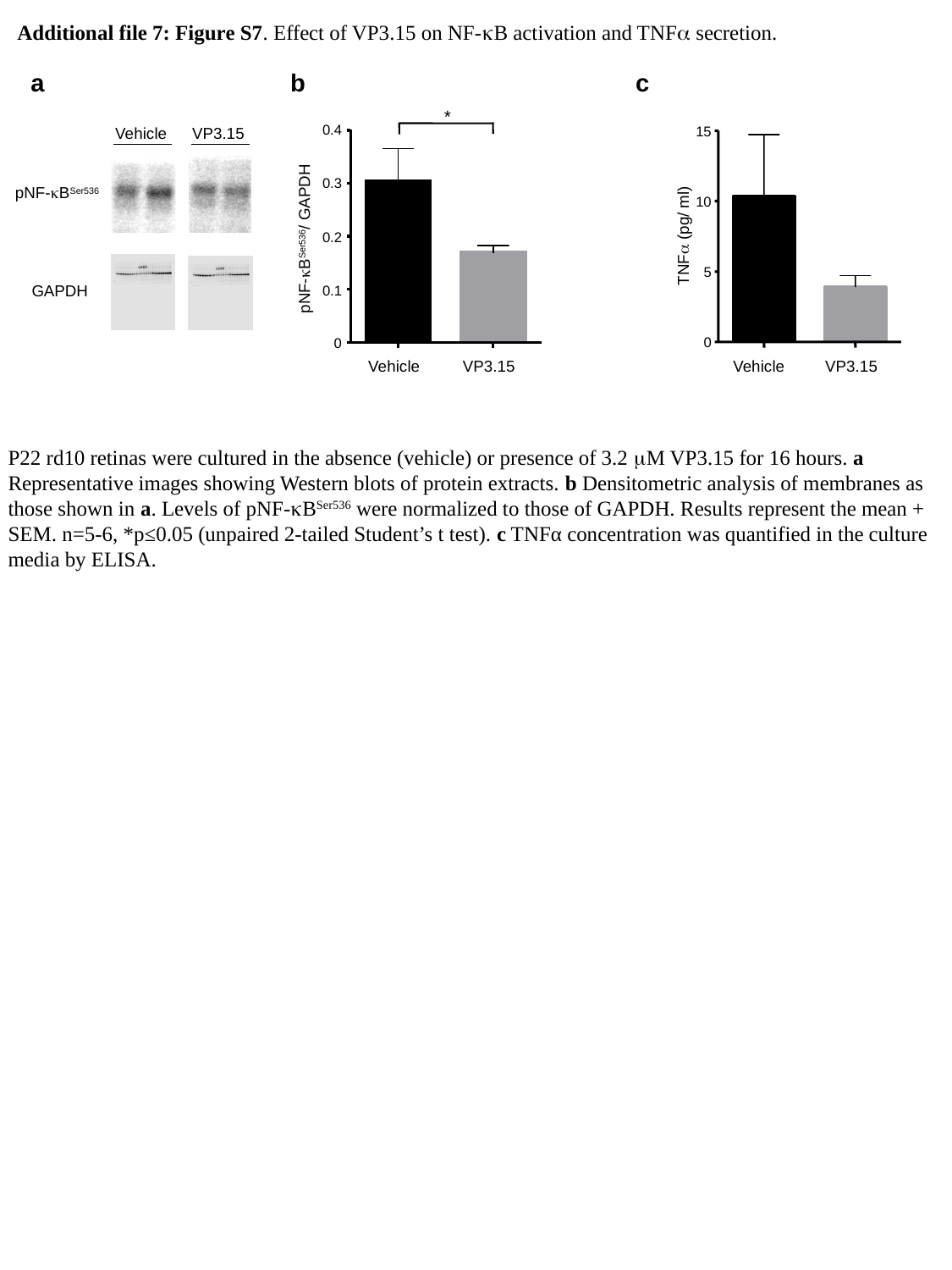

Additional file 7: Figure S7. Effect of VP3.15 on NF-kB activation and TNFa secretion.
a
b
c
*
Vehicle
VP3.15
0.4
15
10
5
0
TNFa (pg/ ml)
Vehicle
VP3.15
0.3
pNF-kBSer536
pNF-kBSer536/ GAPDH
0.2
GAPDH
0.1
0
Vehicle
VP3.15
P22 rd10 retinas were cultured in the absence (vehicle) or presence of 3.2 mM VP3.15 for 16 hours. a Representative images showing Western blots of protein extracts. b Densitometric analysis of membranes as those shown in a. Levels of pNF-kBSer536 were normalized to those of GAPDH. Results represent the mean + SEM. n=5-6, *p≤0.05 (unpaired 2-tailed Student’s t test). c TNFα concentration was quantified in the culture media by ELISA.
