# Supplementary material for: Modulation of GSK-3 provides cellular and functional neuroprotection in the rd10 mouse model of retinitis pigmentosa
Source: Mol Neurodegener. 2018 Apr 16;13:19. doi: 10.1186/s13024-018-0251-y (PMC5902946; doi:10.1186/s13024-018-0251-y)
Supplement: Supplementary file 8 — Additional Methods. (DOCX 20 kb) [file 13024_2018_251_MOESM8_ESM.docx]

**Additional Methods**

**N9 microglia cell culture and determination of β-catenin levels.**

The murine N9 microglial cell line, originally provided by Dr Paola Ricciardi-Castagnoli (Singapore Immunology Network, Agency for Science, Technology and Research, Singapore), was obtained from Dr. Labandeira-García (University of Santiago de Compostela, Santiago de Compostela, Spain). N9 microglial cells were cultured in DMEM supplemented with 5% (v/v) FBS, 2 mM L-Glutamine, 100 U/ml penicillin, and 100 mg/ml streptomycin, and maintained at 37ºC, 95% air, and 5% CO_2_ in a humidified incubator. For analysis, the cells were seeded onto 35-mm culture dishes (75x10^4^ cells / dish), cultured for 24h and then treated with either 0.1% (v/v) DMSO or 10 μM VP3.15 for the indicated time periods. The cells were then processed for immunoblots as described under Methods.

**Retina explant cultures**.

P22 *rd10* were euthanized, and their eyes were enucleated. Retinas were dissected and cultured free-floating in M24 multiwell plates for 16 h in 1 ml R16 medium (Marchena et al. 2017). Retinas were subsequently processed for immunoblots as described under Methods, and the culture medium for ELISA.

**TNFα levels determination.**

TNFα concentration was measured in culture medium from retinal explants by ELISA assay (BioLegend, San Diego, CA, USA) following the manufacturer’s instructions.

**Antibody Table**

| **Antibody** | **Host species** | **Dilution** | **Manufacturer** | **Catalog number** |
| --- | --- | --- | --- | --- |
| β-catenin | Mouse | WB, 1:1000 | BD Biosciences | 610153 |
| pNF-κB^Ser536^ | Rabbit | WB, 1:500 | Cell Signaling | 3033 |
| PKCα | Rabbit | IH, 1:1000 | Sigma | 4334 |
| RBPMS | Rabbit | IH, 1:500 | Abcam | ab194213 |
